# Punicalagin-Loaded Alginate/Chitosan-Gallol Hydrogels for Efficient Wound Repair and Hemostasis

**DOI:** 10.3390/polym14163248

**Published:** 2022-08-09

**Authors:** Jaewon Ju, Jungwoo Kim, Yeonsun Choi, Subin Jin, Sumin Kim, Donghee Son, Mikyung Shin

**Affiliations:** 1 Department of Intelligent Precision Healthcare Convergence, Sungkyunkwan University (SKKU), Suwon 16419, Korea; 2 Center for Neuroscience Imaging Research, Institute for Basic Science (IBS), Suwon 16419, Korea; 3 Department of Biomedical Engineering, Sungkyunkwan University (SKKU), Suwon 16419, Korea; 4 Department of Electrical and Computer Engineering, Sungkyunkwan University (SKKU), Suwon 16419, Korea; 5 Department of Superintelligence Engineering, Sungkyunkwan University (SKKU), Suwon 16419, Korea

**Keywords:** wound-healing material, polyphenol, hemostasis, adhesive hydrogel

## Abstract

For recently devised wound-healing materials, a variety of acute application systems with sustainable therapeutic effects on wound sites have been suggested. For example, hydrogel-type healing agents with porous structures and high drug encapsulation efficiencies have been developed for wound repair. However, challenges remain about the poor mechanical and adhesive properties of hydrogels. Herein, we propose a punicalagin (PC)-containing wound-healing hydrogel in adhesive form that is mechanically stable and has sustainable wound-healing therapeutic efficiency. The APC hydrogel, composed of alginate (ALG), PC, and chitosan–gallol (CHI–G), exhibits significant mechanical and self-healing properties, thus indicating that PC increases cross-linking in ALG/CHI–G as macromolecule. The PC-containing mechanically enhanced hydrogel demonstrates high tissue adhesiveness. Sustainable PC release for 192 h, which indicates high therapeutic effect of the released PC, and great blood compatibility are evaluated based on rapid blood coagulation and minimal hemolysis. The cytocompatibility and wound-healing abilities of the PC-containing APC hydrogel are greater than those of the non-PC hydrogel, as verified by cell compatibility and wound scratch assays. These results indicate that a suitable concentration of PC-containing hydrogel with sustainable moisture condition and PC release may inspire further polyphenol-agent-containing hydrogels as wound-healing agents with structural stability and therapeutic efficiency.

## 1. Introduction

The occurrence of wounds is inevitable in life, whether in civilian, military, or surgical situations. Thus, acute and correct treatments are necessary to avoid infections or other complications, such as osteomyelitis and dermatitis [1]. The wound-healing process comprises four stages: hemostasis, inflammation, granulation, and tissue remodeling [2]. This process is stimulated by various factors, such as growth factors and immune-system factors, that accelerate tissue regeneration and clearance of pathogens. Currently available wound-healing agents that exhibit significant efficiency contain additional molecules, such as growth factors [3], stem cells [4,5], and immunomodulatory factors [6].

One example of such wound-healing agents is the hydrogel type, which provides excellent environments for activating wound-healing derived molecules via moisture and 3D porous structures. Hydrogel-based materials block defect sites and encourage hemostasis by providing appropriate pressure and cooling effects in the initial wound-healing process. Moreover, their high efficiencies at encapsulating drug or therapeutic molecules have been demonstrated by the significant regeneration of tissue [7]. For example, an antioxidant-loaded alginate hydrogel maintains its mechanical properties while gradually releasing antioxidant compounds that improve anti-inflammatory and antibacterial activity in the wound site [8]. However, although hydrogel agents demonstrate excellent wound repair properties in practically every healing process, they also exhibit a variety of disadvantages, including non-long-lasting moisture environment, toxicity, non-biocompatibility of inorganic crosslinking agents [9] or crosslinking mechanism (such as photo crosslinking [10]), and lack of initial hemostasis in the absence of specific blood coagulation factors. Thus, presently, hydrogel agents support the prevention of bleeding exposure through the provision of a physical barrier.

**Figure 1:**
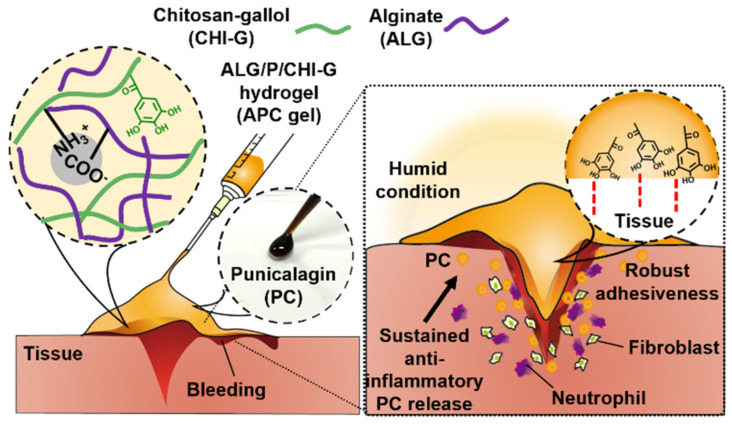
Schematics of the ALG/PC/CHI–G (APC) hydrogel (**left**) with robust tissue-adhesiveness and sustained release of therapeutic PC (**right**) for effective wound healing.

Therefore, this study introduces a new type of wound-healing hydrogel (termed as APC hydrogels) that overcomes the limitation by acting as a highly adhesive and therapeutic material, thereby allowing direct hemostasis and enhanced tissue regeneration in wound sites (Figure 1). For that, punicalagin (PC), a well-known nutritional supplement found in pomegranate (*Punica granatum*) [11,12], is incorporated in the hydrogel. They have high amounts of phenolic compounds which capable of showing anti-cancer, anti-inflammatory, and antioxidant effect [13,14]. In particular, such polyphenols lead to free-radical scavenging effect through reducing reactive oxygen species from cells [15,16]. In addition to that, the phenolic moieties existed in punicalagin can provide high tissue adhesion and blood compatibility to the hydrogels. The polymeric network for overall gelation is formed by electrostatic interactions between gallol-conjugated chitosan (CHI–G) and alginate (ALG)—which are representative biocompatible polysaccharides [17,18]. The gallol groups in CHI-–G encourage robust wet-resistant tissue adhesiveness and blood coagulation, thus accelerating the initial wound-healing process. In addition, highly negative ALG enhances cohesiveness of the hydrogels via ionic attraction with highly positive CHI–G, helping mechanically stable gelation [19,20] and facile loading of the therapeutic molecules, punicalagin. The APC hydrogels consisting of PC, ALG, and CHI–G exhibit topical attachment on the wound sites and acceleration of hemostasis, thus improving initial wound healing process as well as long-term therapeutic effects.

## 2. Materials and Methods

### 2.1. Materials

Chitosan (CHI, 75–85% deacetylated, 50,000–190,000 Da), gallic acid (GA), 1-hydroxybenzotriazole hydrate (HOBt), and alginate (ALG), were purchased from Sigma-Aldrich (St. Louis, MO, USA). Furthermore, 1-ethyl-3-(3-dimethylaminopropyl) carbodiimide hydrochloride (EDC) was obtained from Tokyo Chemical Industry Co., Ltd. (Tokyo, Japan), whereas punicalagin (PC) was purchased from Santa Cruze Biotechnology (Santa Cruz, CA, USA).

### 2.2. Synthesis of Gallol-Conjugated CHI (CHI–G)

The CHI–G was synthesized using the EDC/HOBt coupling reaction [21]. In brief, CHI (500 mg) and HOBt (398.4 mg) were dissolved in 0.05M HCl solution (37.5 mL) and 50% ethanol (12.5 mL), respectively over 1 h, after which GA (251 mg) was dissolved in 50% ethanol (2.5 mL) and added to the CHI/HOBt solution over 30 min. The pH was sequentially adjusted to 3.3–3.8. Subsequently, EDC (565 mg) was dissolved in 10 mL of 50% ethanol, after which the solution was added to the CHI/HOBt/GA mixture, and the reaction was allowed to progress for 4 h (pH approximately 4). To remove the unreacted GA, EDC, and HOBt, the product was dialyzed using a regenerated cellulose membrane (molecular weight cut-off (MWCO) = 3.5 kDa) in NaCl containing deionized water (DW; pH 4.0) for 3 d and DW for 4 h. The final product was obtained via lyophilization.

### 2.3. Preparation of APC Hydrogels

Each polymer was dissolved in DW as follows: 3% (*w*/*v*) ALG, 100% (*w*/*v*) PC, and 3% lyophilized CHI–G (*w*/*v*). For gelation, PC (at a final concentration of 0, 15, 30, or 60 mM) was added to the ALG solution, and subsequently, CHI–G solution was mixed with them using micro-spatula or vortexing vigorously.

### 2.4. Physicochemical Characterization and Rheological of APC Gel

First, the UV-Vis absorbance spectra of the CHI–G was obtained using an Agilent 8453 UV-Vis spectrometer (Agilent 8453; Agilent Technologies, Santa Clara, CA, USA). As a control, lyophilized CHI powder was prepared after dissolving chitosan (500 mg) in 0.05 M HCl (50 mL), adjusting its pH value to 3.5–3.8 and being dialyzed in NaCl containing DW (pH 3.8) for 3 d and DW for 4 h using a regenerated cellulose membrane (molecular weight cut-off (MWCO) = 3.5 kDa). Each lyophilized sample was dissolved in DW with a concentration of 0.05 mg mL^−1^, after which the solutions were put into quartz cuvettes for measurement (A_299_). On the other hand, the degree of gallol conjugation (DOC_Ga_%) of CHI–G were evaluated using UV. The DOC_Ga_% values were determined using a GA standard curve over a concentration range of 0.005–0.05 mg mL^−1^ (y = 0.0001x + 0.0000001, y = absorption value, and x = concentration of CHI–G). Additionally, the turbidities of 2% CHI, 3% CHI, 2% CHI–G, and 3% CHI–G (concentration of 5 mg mL^−1^, DW) were determined based on their A_800_ values; in particular, they were evaluated using a SYNERGY HTX multi-mode microplate reader (BioTek, Winooski, VT, USA). For investigating the gelation mechanism of APC hydrogels, Fourier transform infrared (FT-IR) spectra of lyophilized CHI–G, ALG, ALG/PC, ALG/CHI–G and APC hydrogels distributed in potassium bromide pallets were obtained (TENSOR27, Bruker, Bremen, Germany). In addition, the microporosity of the lyophilized APC hydrogel containing PC (0, 15, 30, and 60 mM) was analyzed by scanning electron microscope (SEM; JEOL Ltd., Tokyo, Japan). The pore size was estimated at randomly chosen four different positions using ImageJ software (Version 1.8.0; National Institutes of Health, Bethesda, MD, USA).

To compare the rheological properties of the APC gels with different orders (15 mM PC contained gel) and different concentrations of PC (0, 15, 30, and 60 mM), a Discovery Hybrid Rheometer 2 (TA Instrument, New Castle, DE, USA) with a 20 mm-diameter parallel plate geometry was used at 25 °C (gap = 100 μm, frequency sweep mode from 0.1 to 10 Hz at 2% strain). The self-healing properties of the APC gels were evaluated using the rheometer based on their performance at an alternating oscillatory strain at a fixed frequency of 1 Hz. Furthermore, the storage and loss moduli were measured based on alternating strains of 0.5% and 1000% at 3 min intervals at 1 Hz. Finally, shear viscosity (Pa·s) was tested at shear rates from 10^−2^ to 10^3^ s^−1^.

### 2.5. Adhesive Evaluation of APC Gel

Adhesiveness was evaluated via a tensile test using a universal testing machine (UTM; Instron 5943, Norwood, MA, USA). Five hundred milligrams (500 mg) of each sample (APC gels with different concentrations of PC) were applied between two sides of rat skin tissue (15 mm × 15 mm; 8–10-wk-old male Sprague Dawley (SD) rats), which was fixed onto a gripper with double-side tape (3M, Saint Paul, MN, USA) for 2 min and stretched at a speed of 20 mm min^−1^.

### 2.6. In Vitro PC Release Test and Degradation Profile Analysis

To demonstrate the PC release kinetics of each APC gel, 100 mg of hydrogel (15 mM and 30 mM of PC) were placed into a 2 mL tube and incubated in 1 mL 1× phosphate-buffered saline (PBS) buffer (pH 7.4) at 37 °C. The absorbance of supernatant was measured at 372 nm using a microplate reader at the following time points: 0.5, 1, 2, 3, 6, 9, 12, 24, 48, 72, 96, 120, 144, 168, and 192 h. The PC standard curve was also established over the concentration ranging from 0.0046 (0.005 mg mL^−1^) to 0.0184 mmol L^−1^ (0.02 mg mL^−1^) (y = 0.0212x + 0.0013), x = absorption value, and y = molar concentration of PC (mmol L^−1^). Degradation profile was evaluated by measuring remained weight of hydrogels in 24-well transwell (SPL Life Science, Pocheon, Korea). APC hydrogels (100 mg; 0 mM, 15 mM, and 30 mM of PC contained) were incubated in 1 mL of PBS (pH 7.4) at 37 °C and their weights were measured at the following time points: 0, 6, 12, 24, 36, 48, 72, 96, and 120 h.

### 2.7. Blood Coagulation Test and Hemolysis Assay

To evaluate the blood coagulation ability and blood compatibility of the APC gels, each gel was prepared in a 2 mL vial (containing 100 mg of 0-, 15-, 30-, or 60-mM PC in APC gel), after which 100 µL of whole rat blood was added to each well. The blood clotting time was evaluated based on its appearance every 30 s and checked after the unclotted blood was washed with saline [22]. Hemolysis assays were performed using whole rat blood. The whole blood was centrifuged at 9000 rpm for 10 min; after the supernatant was removed, red blood cells (RBCs) were washed with saline and then diluted to a final concentration of 10% (*v*/*v*) using 1× PBS buffer. Diluted RBCs (500 µL) and each hydrogel formulation (0-, 15-, 30-, or 60-mM PC-containing APC hydrogel) (100 µL) were mixed and incubated at 37 °C for 1 h. After incubation, the samples were centrifuged at 3500 rpm for 15 min, and the supernatant was collected for evaluation. The optical density (OD) values of each sample were measured using a microplate reader at 540 nm, and the degrees of hemolysis (%) were calculated as:

Hemolysis (%) = (OD of sample − OD of negative control)/(OD of positive control − OD of negative control) × 100

where positive control and negative control represent Triton-X (Sigma-Aldrich, St. Louis, MO, USA) and saline, respectively.

### 2.8. In Vitro Cytocompatibility and Wound Scratch Test

In vitro cytocompatibility tests were performed on the APC hydrogel using fibroblast cells (L929 cell line). The cells were cultured in 24-well plates with a cell density of 2.5 × 10^4^ for 24 h. The hydrogels (100 mg; 0, 15, 30, and 60 mM PC) were incubated in 1 mL of PBS buffer (pH 7.4) at 37 °C. After 24 h, 50 μL of supernatant in hydrogel solution were added. Cell viability was evaluated via fluorescence microscopy after 24 h using a live/dead assay (Thermo Fisher Scientific, Waltham, MA, USA). The number of live or dead cells was calculated using ImageJ software.

Next, wound scratch assay was conducted using L929 cells in 24-well plates. The cells were cultured for 24 h at a cell density of 2.5 × 10^4^, and monolayer cells were scratched using scratch tips (5 mm width scratch). The cells were washed with 1× Dulbecco’s PBS (DPBS) to remove the scratched cells, after which the cells were incubated with the pre-formed supernatant of the hydrogel solutions (0, 15, and 30 mM PC). The wound-scratched cell layer was imaged at 0, 24, and 48 h using fluorescence microscopy with live/dead assay agents. The wound-healing areas were analyzed using ImageJ software. Relative wound closure percentage was estimated as follows: Relative wound closure [%] = wound closure area (pixel) × 100 (%/initial wound area (pixel) [23].

## 3. Results and Discussion

### 3.1. Preparation of APC Hydrogels

**Figure 2:**
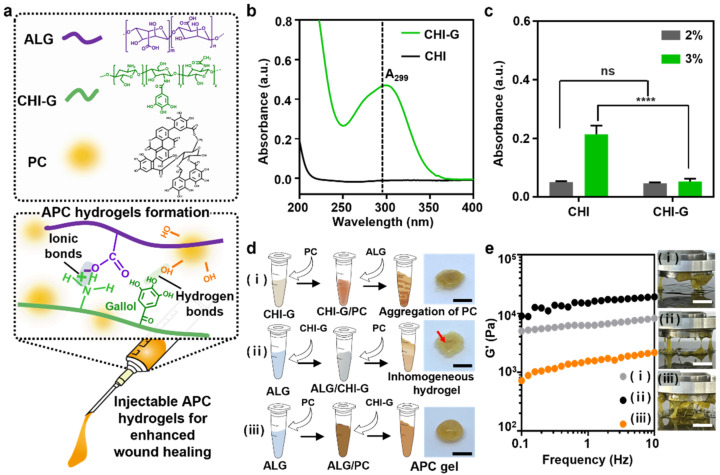
Preparation of APC hydrogels. (**a**) Chemical structure of the main components existed in injectable APC hydrogels (**top**) and their gelation via ionic and hydrogen bonds (**bottom**). (**b**) UV-Vis spectra of CHI (black) and CHI–G (green) (*n* = 3). Dashed line (black) indicates 299 nm. Statistical significance and *p* values are determined by one-way ANOVA followed by Tukey’s post-hoc test. **** *p* < 0.0001; ns denotes not significant. (**c**) Turbidities of different concentrations of CHI (gray) and CHI–G (green) solubilized in water. (**d**) Schematic of gelation condition for varying mixing sequences of components ALG, PC, and CHI–G. Red arrow indicates aggregation of PC. Scale bar: 10 mm. (**e**) Rheological properties (**left**) and photos (**right**) of different hydrogels for varying sequences. Scale bar: 10 mm.

APC hydrogels were prepared via mixing of ALG, CHI–G, and PC solution. The gallol groups of CHI–G provides robust tissue-adhesion on curved tissue surfaces against bleeding, thus allowing initial hemostasis and subsequent wound healing [24,25]. ALG forms a hydrogel upon mixing with CHI–G, which is based on ionic crosslinking. The hydrogels have high mechanical stability even in physiological conditions (Figure 2a) [26]. For CHI–G, the gallol conjugation was confirmed in UV-Vis spectra of the soluble polymer when compared to that of relatively soluble CHI with protonated amine groups. The absorption peak of CHI–G at 299 nm indicated gallol modification, based on a comparison with the gallol peak at 260 nm. Gallol-tethered chitosan exhibited increased length of delocalized electrons, thereby indicating a red-shifted peak. The absorption peak at 299 nm revealed the degree of gallol conjugation to be 15.7 ± 1.0% (Figure 2b). For strong adhesion of the final hydrogel, high concentration of polymeric components was required. The mixtures based on the higher concentrations of components demonstrated suitable hydrogel characteristics, specifically long-lasting gel viscosities after wound adhesion; therefore, 3% (*w*/*v*) of ALG and CHI–G were applied in subsequent formulations. The mixture based on 3% dissolved CHI–G demonstrated a lower turbidity (0.05 ± 0.03) than that exhibited by the mixture based on unconjugated CHI (0.2 ± 0.02) [27] (Figure 2c). Gallol conjugation produces decreased crystallinity in CHI and increased solubility even in aqueous solutions. The highly soluble CHI–G enables homogenous APC gelation and results in overall crosslinking with ALG than that produced by CHI. In our hydrogel system, the mixing order was a considerable part of minimizing aggregation to form a homogenous hydrogel (Figure 2d). The first hydrogel was concocted via the addition of ALG to a CHI–G/PC solution. For the second hydrogel, PC extracts were mixed with the previously formed ALG/CHI–G solution. Both hydrogels were heterogenous, and, particularly for the hydrogel with PC extracts, aggregation was easily observed. In contrast, for the third hydrogel, CHI–G was added to an ALG/PC solution because adding the CHI–G last was expected to promote stable ionic crosslinking by minimizing interruptions to PC molecules in the ALG solution. Each synthesis method exhibited different rheological properties. The first and second methods produced hydrogels that exhibited high G′ values: 5894 ± 1522 Pa and 13,610.5 ± 2336.2 Pa, respectively (Figure 2e). These high values indicated aggregation within the non-homogenous hydrogel, as shown in the figure. By contrast, the third method produced a homogenous and viscous hydrogel (1604 ± 352 Pa), enabling its use as a stable and well-conjugated material. Therefore, the proposed APC hydrogel exhibited uniform PC content with tissue-adhesive gallol moieties. The components of the APC hydrogel have the potential to maintain a wound-healing hydrogel condition throughout the healing process.

### 3.2. Physicochemical Characterization and Rheological Properties of APC Hydrogel

**Figure 3:**
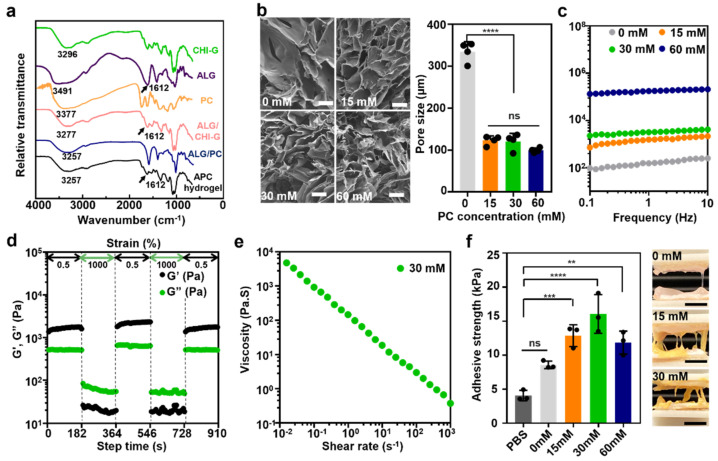
Physicochemical characterization and rheological properties of APC hydrogels. (**a**) FT-IR spectra of CHI–G (green), ALG (violet), PC (orange), ALG/CHI–G (pink), ALG/PC (blue) and APC hydrogel (black). (**b**) Cross-sectional SEM images of the APC hydrogels containing PC with different concentrations (0, 15, 30, and 60 mM) (**left**) and their pore sizes (**right**) (*n* = 4). Scale bar: 100 µm. (**c**) Frequency sweep-storage (G′) moduli at 2% strain, 25 °C of 0 mM (gray), 15 mM (orange), 30 mM (green), and 60 mM (blue) PC-containing APC hydrogels. (**d**) Self-healing properties and (**e**) shear-thinning performance of 30 mM APC hydrogel. (**f**) Adhesive strengths of hydrogels with different PC concentrations. (*n* = 3) Scale bar: 10 mm. All statistical significance and *p* values are determined by one-way ANOVA followed by Tukey’s post-hoc test. **** *p* < 0.0001; *** *p* < 0.001, ** *p* < 0.01; ns denotes not significant.

The APC hydrogels were prepared based on different concentrations of PC (0 mM (ALG/CHI–G hydrogel), 15 mM, 30 mM, and 60 mM) to demonstrate the effects of PC in providing structural stability to the hydrogel and on its sustainable release as a therapeutic agent. First, the driving force for APC hydrogels was demonstrated by Fourier-transform infrared (FT-IR) analysis (Figure 3a). As mentioned in Figure 2d, the mixing order of three components for APC gelation was important. The ALG and PC should be firstly mixed, and then CHI–G should be added to the mixture of ALG/PC for homogenous gelation. This means that each component can have intermolecular interactions in the mixing procedures. In the FT-IR spectra of ALG/PC mixture (Figure 3a, blue), the O-H vibration peak was lower-shifted to 3257 cm^−1^ when compared to those of ALG (3491 cm^−1^) (violet) or PC alone (3377 cm^−1^) (orange), indicating formation of hydrogen bonds in-between ALG and PC [28]. Such shift (3491 cm^−1^ for ALG alone 3277 cm^−1^) was also detected in the spectra of the mixture of ALG/CHI–G (pink), showing the hydrogen bonds between those polymers. Furthermore, the carboxyl peak appeared at 1612 cm^−1^ in the spectra of ALG disappeared in both spectra of ALG/CHI–G mixture (Figure 3a, pink) and APC hydrogel (black), meaning electrostatic interactions of those polymers [29]. Second, APC hydrogels had microporous structures depending on the PC amounts incorporated (Figure 3b). Although the pore size of ALG/CHI–G hydrogels without PC (e.g., 0 mM) was 334.3 ± 23.7 µm, those of APC hydrogels were 123.0 ± 10.8 µm for 15 mM, 120.5 ± 19.3 µm for 30 mM, and 99.6 ± 5.5 µm for 60 mM, showing denser structure with smaller pores. The rheological properties also indicated the relationship between the PC concentration and the crosslinking density and gelation degree; in particular, the G′ value increased as PC content increased (Figure 3c). For example, the 0 mM (460.8 ± 66.8 Pa), 15 mM (1604 ± 352 Pa), 30 mM (3370.6 ± 973.2 Pa), and 60 mM (143,065 ± 28,195.9 Pa) hydrogels showed an increase in G′ value with respect to the concentration of PC. These results demonstrated that PC, a large polyphenol component with high molecular weight, has a higher storage modulus, which not only plays a therapeutic role in wound healing, but also has a great effect on stabilizing the properties of the APC hydrogel [30]. Considering previous studies, the ALG/CHI hydrogels formed by the only ionic crosslinking have a limitation of weak mechanical properties. When incubated under physiological condition (pH 7.4), they show rapid dissociation. To improve this, small crosslinkers to trigger additional covalent bonds in the hydrogel network can be used, yet such crosslinker has inevitable toxicity [31]. In that view, the incorporation of PC into the polysaccharide network can be useful strategy for increasing their mechanical stability. Among the synthesized hydrogels, the 30 mM PC-containing APC hydrogel was selected for the estimation of the rheological properties of the APC hydrogel, demonstrating self-healing performance at oscillatory strains between 0.5% and 1000% for 3 min with a fixed frequency of 1 Hz (Figure 3d). The recovery of the G′ value revealed the applicability of the hydrogel to tough tissue surfaces or internal tissue injuries due to the sustainable crosslinking network of the APC hydrogel [32]. The hydrogel also exhibited in situ shear-thinning gelation, as evidenced by the decrease in shear viscosity with respect to increases in the shear rate. This verified its nature as a useful therapeutic hydrogel agent that can be applied via simple injection at injury sites (Figure 3e). Furthermore, the adhesive strengths of the APC hydrogels with different PC concentrations were compared on freshly excised rat skin (Figure 3f). The PC-containing APC hydrogels demonstrated high adhesive performance. For example, the 15 mM PC-containing APC hydrogel exhibited an adhesive performance of 12.9 ± 1.6 Pa, whereas the 30 mM PC-containing APC hydrogel had 16.1 ± 2.8 Pa. However, the 60 mM PC-containing APC hydrogel exhibited a lower G′ value than that of the 30 mM hydrogel, indicating that excessive amounts of PC decrease the adhesive properties of the APC hydrogel, which, in turn, increases its cohesiveness.

### 3.3. PC Release and Degradation Kinetics of the APC Hydrogels for Therapeutic Wound Healing Effects

**Figure 4:**
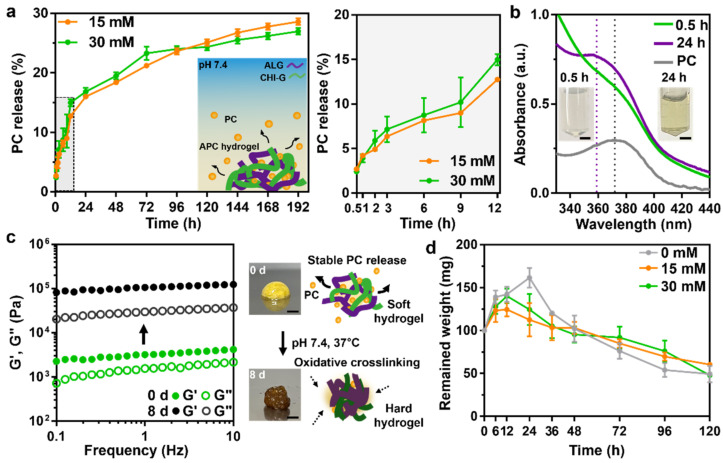
(**a**) PC release kinetics of 15 mM (orange) and 30 mM (green) APC hydrogels (**left**) and their initial release profile observed within 12 h (**right**) (*n* = 3). (**b**) UV-Vis spectra of the released eluents from the hydrogels at 0.5 h (green) and 24 h (violet) and PC alone (gray), and photos of their eluents. Scale bar: 5 mm. The dashed lines for the wavelength of 359 nm and 372 nm. (**c**) Rheological properties of the APC hydrogels with 30 mM incubated for 0 (green) or 8 d (black). Filled symbols for storage modulus (G′) and open symbols for loss modulus (G″). The photos indicate colorimetric changes of the APC hydrogels during incubation and schematic illustration shows oxidation of PC as time went by. Scale bar: 5 mm. (**d**) Degradation profile of APC hydrogels (0 mM (gray), 15 mM(orange), and 30 mM (green)) (*n* = 3). All statistical significance and *p* values are determined by one-way ANOVA followed by Tukey’s post-hoc test.

APC hydrogel system could be an efficient drug delivery platform for wound healing. According to recent studies, PC, which had long been used as a traditional medicine, is now commonly known for its high anti-bacterial and anti-inflammatory activity [33,34]. We thus observed the PC release kinetics of the APC hydrogels with different concentrations (Figure 4a). The released PC was evaluated every 24 h for 192 h during incubation at 37 °C with PBS buffer; rapid release was observed for the first 12 h (Figure 4a, right graph), whereas stable release was observed after 24 h. Such initial burst release of PC might be due to rapid swelling of the hydrogels. The degree of PC release was estimated using UV-Vis spectra, where a 359 nm PC absorption peak emerged (violet, Figure 4b) after 24 h release, which also indicated a shift from the 372 nm for the PC alone (gray, Figure 4b). An increased release rate of PC with respect to increased concentration of PC in the APC hydrogel was observed for the first 72 h, where the 15 mM hydrogel demonstrated a higher release rate (approximately 21.1 ± 0.26%) than that exhibited by the 30 mM hydrogel (approximately 23.8 ± 1.1%). However, after 72 h release, the PC release of the 15 mM hydrogel became higher than that of the 30 mM hydrogel and finally achieved a value of approximately 28.6 ± 0.56% after 192 h. We also estimated the rheological properties of the releasing hydrogel after 192 h: the G′ values of the 15 mM (29,855.4 ± 3295 Pa) and 30 mM (109,131.3 ± 8204 Pa) hydrogels both had high rates of increase, thereby revealing that their crosslinking densities increased across time (Figure 4c). As expected, the G′ value of the APC hydrogels via an ionic crosslinking between ALG and CHI–G increased as time went by. This was due to additional oxidative crosslinking by PC incorporated. When large amounts of PC were included, the internal crosslinking, together with the oxidation of gallol and PC, increased with time, and PC release was limited by the rigid hydrogel. Based on these results, the PC-containing APC hydrogel with high adhesiveness clearly has potential for application in defect wound sites, thus providing a promise of therapeutic healing via PC release. In addition to that, the wound healing hydrogels should generally provide moisture on the injury site and possess therapeutic effects. Thus, it is important to evaluate resorption rate of the hydrogels. As shown in Figure 4d, the APC hydrogels (e.g., 15 mM or 30 mM) exhibited slow resorption rate over 120 h (remained weight = 60.5 ± 1.4 mg of 15 mM and 54.1 ± 13 mg of 30 mM at 120 h) compared to that of ALG/CHI–G hydrogel alone (49.4 ± 9.6 mg) at 120 h). The result might be also because PC incorporated in the hydrogel forms additionally covalent crosslinking.

### 3.4. Blood Compatibility of APC Hydrogel

**Figure 5:**
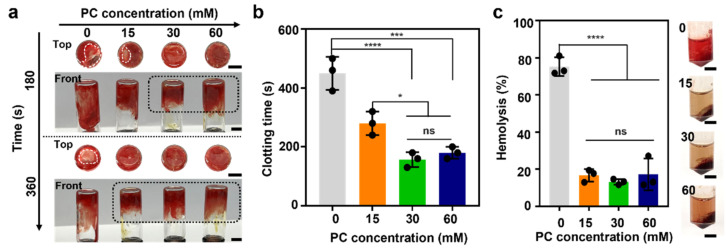
All statistical significance and *p* values are determined by one-way ANOVA followed by Tukey’s post-hoc test. Blood compatibility of APC hydrogel. (**a**) Blood coagulation assays and (**b**) coagulation times of 0 mM(gray), 15 mM (orange), 30 mM (green), and 60 mM (blue) PC-containing APC hydrogels. Black and white dashed lines indicate clotting and non-clotting performance, respectively. Scale bar: 5 mm. *n* = 3. (**c**) Hemolytic activity evaluation of 0 mM (gray), 15 mM (orange), 30 mM (green), and 60 mM (blue) PC–containing APC hydrogels. (*n* = 3) Scale bar: 5 mm. All statistical significance and *p* values are determined by one-way ANOVA followed by Tukey’s post-hoc test. * *p* < 0.05, *** *p* < 0.001, **** *p* < 0.0001; ns denotes not significant.

Acute blood coagulation and hemostasis are the first stages of the wound-healing process. Wound repair starts at immediate hemostasis; subsequently, blood clotting cascade, with plate aggregation and clot formation, occurs around the wound site, thus forming an inflammatory activated condition [35,36]. To demonstrate the blood compatibility of the APC hydrogel, two evaluation tests were conducted on different concentrations of PC-containing hydrogels and fresh rat blood. First, blood coagulations were tested using whole blood. The PC-containing APC hydrogels demonstrated distinct blood clotting abilities within 280 s, showing blood clotting at the bottoms of the vials (Figure 5a); for comparison, blood coagulation for the 0 mM APC hydrogel was estimated at 450 ± 56 s (Figure 5b). As the PC concentration was increased, the amount of time required for blood clotting became shorter: for example, the 15 mM and 30 mM hydrogels required 280 ± 40 s and 156.7 ± 25 s, respectively, for blood clotting. The 60 mM APC hydrogel demonstrated a similar blood-clotting time (180 ± 20 s) to that of the 30 mM hydrogel. The PC components of the APC hydrogel with high G′ values maintained viscous gel properties due to additional physical interactions between PC, ALG, and CHI–G and optimization of the internal structure to blood clotting.

Hemolysis tests were performed to evaluate the APC hydrogels for any negative reactions toward RBCs (Figure 5c). Herein, RBCs were incubated with the APC hydrogel and analyzed based on the supernatant of the incubated solution. The PC-containing APC hydrogel exhibited improved blood compatibility compared to that of the 0 mM APC hydrogel (75.2 ± 5.1%), which indicates a high hemolysis level. Moreover, the 15 mM (16.7 ± 3.3%), 30 mM (13.1 ± 1.5%), and 60 mM (17.2 ± 8.6%) hydrogels demonstrated no significant differences with respect to PC concentration. Based on these results taken together, the APC hydrogel, which exhibits high blood compatibility due to increase in the physical properties of hydrogels with PCs, is also involved in hemostasis and coagulation. Thus, it promises a crucial role in the wound healing of acute injuries.

### 3.5. Cell Viability and In Vitro Wound Scratch Assay

**Figure 6:**
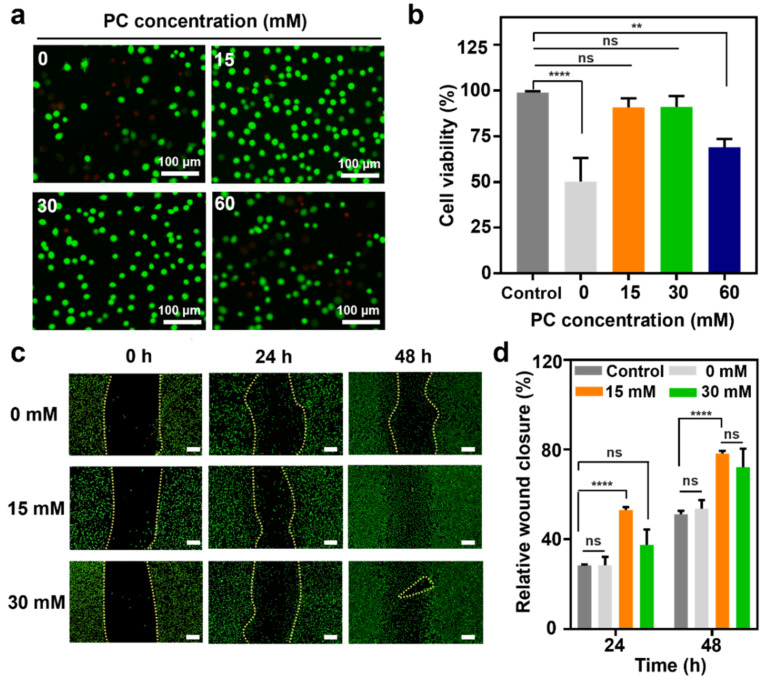
In vitro cell viability and wound scratch assay. (**a**,**b**) Cell viability test. Scale bar: 100 µm. (*n* = 3). (**c**) Wound scratch assay and wound closure for 0 mM, 15 mM, and 30 mM PC-containing APC hydrogels. Scale bar: 200 µm. Yellow lines indicate scratched area. (**d**) Wound-healing percentage (*n* = 3). All statistical significance and *p* values are determined by one- and two-way ANOVA followed by Tukey’s post-hoc test. ** *p* < 0.01, **** *p* < 0.0001; ns denotes not significant.

Previous studies have demonstrated antioxidant activity of PC extracts by preventing reactive oxygen species (ROS) formation or regulating ROS combating enzyme [37]. In addition, PC can improve fibroblast migration and proliferation on skin wound with minimal inflammatory and angiogenic effects, initially promoting wound healing responses and ultimately remodeling extracellular matrix (ECM) [38,39]. To evaluate the cytocompatibility of the APC hydrogel as an effective wound-healing material with therapeutic effects of PC, an in vitro viability test was conducted using a live/dead assay. APC hydrogels with different PC concentrations were kept in a physiological buffer for 1 d and then added to cell membranes. Almost all PC-containing APC hydrogels exhibited high viability (approximately 91.1%); in particular, the 15 mM (90.9 ± 4.79%) and 30 mM (91.1 ± 5.89%) APC hydrogels demonstrated high biocompatibilities, supported by the non-toxicity of PC, compared with that of the 0 mM APC hydrogel (50.2 ± 12.8%) (Figure 6a,b). Moreover, the 60 mM APC hydrogel exhibited a relatively low viability (68.9.2 ± 4.6%), thus indicating that excessive PC had insignificant effects on biocompatibility.

Wound-healing performance is another important ability that should be present in the hydrogel. In particular, the material should be able to promote the migration of fibroblasts to the injury site and initiate the proliferative phase for wound repair [40]. Thus, a wound-healing effect of the APC hydrogel was investigated for scratches in the fibroblast layer, and the percentages of wound closure were evaluated every 24 h (Figure 6c). The 15 mM APC hydrogel demonstrated significant wound coverage ability (53.1 ± 1.3%) after 24 h compared with those exhibited by control (28.3 ± 0.4%) and 0 mM (28.4 ± 3.9%), and almost complete recoveries were observed for the 15 mM (78.1 ± 1.3%) and 30 mM (72.1 ± 8.2%) hydrogels (Figure 6d). For further discussion, APC hydrogel with 15 mM PC showed slightly higher wound closure abilities than the ones with 30 mM. The result might be because continuous exposure of high concentration of PC to the cells decreases their migration and wound repopulation [41]. Therefore, as a therapeutic wound-healing material, the optimal concentration of PC incorporated in APC hydrogel is critical for full wound repair.

## 4. Conclusions

Herein, an APC wound-healing hydrogel was developed to facilitate the overall wound-healing process. It was determined that the mixing sequence of the components (ALG, PC, and CHI–G) is highly relevant to the formation of a homogenous hydrogel, wherein PC is well dispersed in the ionically crosslinked hydrogel. The mechanically enhanced hydrogel due to PC content exhibited high tissue adhesiveness, which is necessary for maintaining humid conditions on wound sites. The hydrogel demonstrated sustained PC release for 192 h, indicating that the hydrogel can produce promising therapeutic effects via the released PC. High blood compatibility was also verified based on rapid blood coagulation and minimal hemolysis performance. The PC-containing APC hydrogel exhibited higher performance in cell compatibility and wound scratch assays than that exhibited by the ALG/CHI I–G hydrogel without PC. However, excessive PC in the hydrogel decreased its therapeutic effects as a wound-healing material. These results proved that a hydrogel containing a suitable concentration of PC produces an optimal hydrogel system that creates sustainable moisture conditions, enables PC release, and exhibits adhesive properties. Thus, we anticipate that natural polyphenol-agent-containing hydrogels may be potential wound-healing hydrogels via their improved structural stabilities and therapeutic efficiencies.

## Data Availability

The data presented in this study are available in the article.
